# Kv7 voltage-activated potassium channel inhibitors reduce fluid resuscitation requirements after hemorrhagic shock in rats

**DOI:** 10.1186/s12929-017-0316-1

**Published:** 2017-01-17

**Authors:** Sean P. Nassoiy, Kenneth L. Byron, Matthias Majetschak

**Affiliations:** 1 Burn and Shock Trauma Research Institute, Department of Surgery, Loyola University Chicago, Stritch School of Medicine, 2160 S. 1st Avenue, Maywood, IL 60153 USA; 2 Department of Molecular Pharmacology and Therapeutics, Loyola University Chicago, Stritch School of Medicine, 2160 S. 1st Avenue, Maywood, IL 60153 USA

**Keywords:** Linopirdine, Retigabine, Hemorrhagic shock, Hemodynamics, Blood pressure, Resuscitation fluid

## Abstract

**Background:**

Recent evidence suggests that drugs targeting Kv7 channels could be used to modulate vascular function and blood pressure. Here, we studied whether Kv7 channel inhibitors can be utilized to stabilize hemodynamics and reduce resuscitation fluid requirements after hemorrhagic shock.

**Methods:**

Anesthetized male Sprague-Dawley rats were instrumented with arterial and venous catheters for blood pressure monitoring, hemorrhage and fluid resuscitation. Series 1: Linopirdine (Kv7 channel blocker, 0.1–6 mg/kg) or retigabine (Kv7 channel activator, 0.1–12 mg/kg) were administered to normal animals. Series 2: Animals were hemorrhaged to a MAP of 25 mmHg for 30 min, followed by fluid resuscitation with normal saline (NS) to a MAP of 70 mmHg until t = 75 min. Animals were treated with single bolus injections of vehicle, linopirdine (1–6 mg/kg), XE-991 (structural analogue of linopirdine with higher potency for channel blockade, 1 mg/kg) prior to fluid resuscitation. Series 3: Animals were resuscitated with NS alone or NS supplemented with linopirdine (1.25–200 μg/mL). Data were analyzed with 2-way ANOVA/Bonferroni post-hoc testing.

**Results:**

Series 1: Linopirdine transiently (10–15 min) and dose-dependently increased MAP by up to 15%. Retigabine dose-dependently reduced MAP by up to 60%, which could be reverted with linopirdine. Series 2: Fluid requirements to maintain MAP at 70 mmHg were 65 ± 34 mL/kg with vehicle, and 57 ± 13 mL/kg, 22 ± 8 mL/kg and 22 ± 11 mL/kg with intravenous bolus injection of 1, 3 and 6 mg/kg linopirdine, respectively. XE-991 (1 mg/kg), reduced resuscitation requirements comparable to 3 mg/kg linopirdine.

Series 3: When resuscitation was performed with linopirdine-supplemented normal saline (NS), fluid requirements to stabilize MAP were 73 ± 12 mL/kg with NS alone and 72 ± 24, 61 ± 20, 36 ± 9 and 31 ± 9 mL/kg with NS supplemented with 1.25, 6.25, 12.5 and 200 μg/mL linopirdine, respectively.

**Conclusions:**

Our data suggest that Kv7 channel blockers could be used to stabilize blood pressure and reduce fluid resuscitation requirements after hemorrhagic shock.

## Background

In the United States trauma is the 5^th^ leading cause of death in the overall population and the leading cause of death among those aged 5–44 [1]. Hemorrhagic shock is the major cause of potentially preventable death after accidental injuries and accounts for over 40% of deaths within the first 24 h in trauma patients [2]. Adequate fluid resuscitation to compensate for intravascular volume deficits and to support organ perfusion is an essential cornerstone in the treatment of patients with traumatic-hemorrhagic shock [3]. High-volume fluid resuscitation, however, carries the well-recognized risk of fluid overload, which can lead to third-spacing of fluids into tissues, edema formation, coagulopathy, abdominal compartment syndrome, or acute lung injury, and significantly contributes to mortality and morbidity in critically ill patients [4–6]. Furthermore, in patients who fail to meet blood pressure targets during fluid resuscitation, vasopressors are often added at the discretion of the health care provider [5]. Vasopressors, however, can have significant adverse effects and their use is limited by vasoconstrictor-induced ischemia. Although pressure-support resuscitation of hemorrhagic shock with arginine vasopressin (aVP) has been discussed as a possible strategy to improve outcomes [7, 8], drugs which stabilize cardiovascular function, reduce resuscitation fluid requirements and lack significant intrinsic vasopressor activity are not available. Such drugs, however, are highly desirable as they have the potential to reduce morbidity and mortality associated with high-volume fluid resuscitation and vasopressor treatment.

Kv7 voltage-activated potassium channels are important regulators of the membrane potential in excitable cells, such as neurons, cardiomyocytes or smooth muscle cells [9–12]. The expression pattern of Kv7 channels is very similar across species, with Kv7.1, Kv7.4 and Kv7.5 being ubiquitously expressed in every arterial bed so far examined [10].

Kv7 channels display an activation threshold near the resting membrane potential and generate outwardly rectifying potassium currents (M-current), which stabilize resting membrane potential and suppress cell excitability [10].

Preclinical and clinical drug development has so far focused on the therapeutic potential of Kv7 channel modulators in neurological diseases. Several drugs targeting Kv7 channels have been developed. Flupirtine, a Kv7 channel activator, has been approved by the European Medicines Agency as a non-opioid analgesic more than 25 years ago [13]. The Kv7 channel activator retigabine (also known as ezogabine), a structural analog of flupirtine, received approval by the US Food and Drug Administration and the European Medicines Agency for the treatment of partial-onset seizures in adults [14]. Furthermore, the Kv7 channel inhibitor linopirdine has been tested as a cognition-enhancing drug in Alzheimer’s disease, but failed to demonstrate clinically meaningful improvements of cognitive function [15].

More recently, Kv7 channels have been recognized as important regulators of vascular smooth muscle function. Several lines of evidence suggest that drugs targeting Kv7 channels could be useful to modulate vascular reactivity and blood pressure in various pathological conditions [10, 16]. While symptomatic hypotension has been reported as an adverse event after oral administration of high doses of retigabine in phase I clinical trials [17], retigabine has demonstrated the beneficial effect of reducing acute hypertension induced by co-administration of angiotensin II plus arginine vasopressin in rats [18]. Furthermore, the Kv7 channel activator flupirtine has been reported to reduce pulmonary hypertension in rodent models [19, 20]. Whether Kv7 channel blockade could be useful to improve hemodynamics and stabilize blood pressures in hypotensive disease processes, however, remains unknown. Thus, we performed a pilot study to assess the effects of Kv7 channel modulators during fluid resuscitation after hemorrhagic shock in a Wigger’s model of fixed-pressure hemorrhage in rats.

## Methods

All procedures were performed according to National Institutes of Health Guidelines for Use of Laboratory Animals and were approved by the Institutional Animal Care and Use Committee of Loyola University Chicago. Male Sprague-Dawley rats (300–350 g) were purchased from Harlan. Rats were anesthetized with 1.5% isoflurane/100% oxygen. At this dose, rats did not respond to noxious stimuli, but were able to breathe spontaneously. The femoral artery was cannulated with a 22-gauge angiocatheter for arterial blood pressure monitoring and blood withdrawal. The femoral vein was cannulated with 1.5-french tubing for fluid and drug administration. After instrumentation, 5–15 min of stable blood pressure recordings were obtained. Core body temperature was maintained using warming lamps. The following series of experiments were then performed:

- Series 1: Administration of Kv7 channel modulators in normal animals.
- In a first set of experiments, animals (*n* = 3) received 5 intravenous bolus injections of increasing doses of linopirdine (Tocris Bioscience, Bristol, UK, 0.1 – 6 mg/kg in 0.5 mL of NS). In a second set of experiments, animals (*n* = 3) received 6 intravenous bolus injections of increasing doses of retigabine (Alomone Labs, Jerusalem, Israel, 0.1–12 mg/kg in 0.5 mL of NS) followed by an intravenous bolus injection of 6 mg/kg linopirdine (in 0.5 mL of NS) in 15 min intervals. Heart rates (HR), systolic arterial blood pressures (SBP), diastolic arterial blood pressures (DBP) and mean arterial blood pressures (MAP) were recorded in 10 s - 1 min intervals. At the end of the experiments, animals were euthanized (isoflurane inhalation, bilateral pneumothorax).

- Series 2: Bolus administration of Kv7 channel inhibitors during resuscitation from hemorrhagic shock.
- We utilized a Wiggers model of fixed-pressure hemorrhage, as described in detail previously [21]. In brief, animals were hemorrhaged to a MAP of 25 mmHg for 30 min. At t = 30 min, animals were resuscitated with NS until MAP returned to 70 mmHg. MAP was then maintained at 70 mmHg by continuous fluid administration for a total of 45 min, as required. At the end of the resuscitation period animals were euthanized (isoflurane inhalation, bilateral pneumothorax). In a first set of experiments animals received an intravenous bolus injection of vehicle (0.5 mL NS, *n* = 4) or 1 mg/kg (*n* = 5), 3 mg/kg (*n* = 3) or 6 mg/kg (*n* = 3) linopirdine in 0.5 mL NS at the beginning of fluid resuscitation (t = 30 min). In a second set of experiments animals received an intravenous bolus injection of vehicle (0.5 mL NS, *n* = 3) or XE-991 (Alomone Labs, 1 mg/kg in 0.5 mL NS, *n* = 3) at the beginning of fluid resuscitation (t = 30 min).

- Series 3: Linopirdine-supplementation of resuscitation fluid.
- Animals were resuscitated with NS alone (*n* = 4) or with NS supplemented with 1.25 μg/mL (*n* = 4), 6.25 μg/mL (*n* = 3), 12.5 μg/mL (*n* = 3) or 200 μg/mL (*n* = 6) of linopirdine. SBP, DBP, MAP, blood volumes hemorrhaged and resuscitation fluid requirements were recorded in 1 min intervals until t = 30 min and then in 5 min intervals until the end of the experiment. At the end of the experiments, animals were euthanized (isoflurane inhalation, bilateral pneumothorax) and gross necropsies were performed.

### Data analyses and statistics

Data are described as mean ± standard deviation (SD). Data were analyzed with two-way repeated measures (mixed model) analysis of variance and Bonferroni post-hoc tests to correct for multiple testing, as appropriate. A two-tailed *p* < 0.05 was considered significant. All data were analyzed using the GraphPad-Prism 6 software.

## Results

**Fig. 1:**
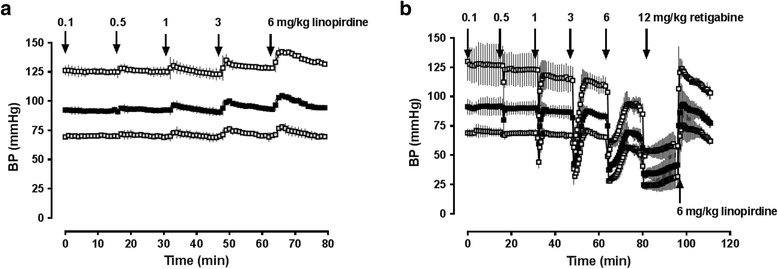
Effects of Kv7 channel modulators on blood pressure in normal rats. **a**. Intravenous injection of increasing doses of linopridine (0.1-6 mg/kg in 0.5 mL normal saline). *Arrows* indicate time points of drug injection. *Open squares*: Systolic blood pressure. *Grey squares*: Diastolic blood pressure. *Black Squares*: Mean arterial blood pressure. BP: Blood pressure (mmHg). *N* = 3. Data are mean ± SD. **b**. Intravenous injection of increasing doses of retigabine (0.1-12 mg/kg in 0.5 mL normal saline) followed by an intravenous injection of 6 mg/kg linopirdine in 0.5 mL normal saline. *Open squares*: Systolic blood pressure. *Grey squares*: Diastolic blood pressure. *Black Squares*: Mean arterial blood pressure. BP: Blood pressure (mmHg). *N* = 3

**Fig. 2:**
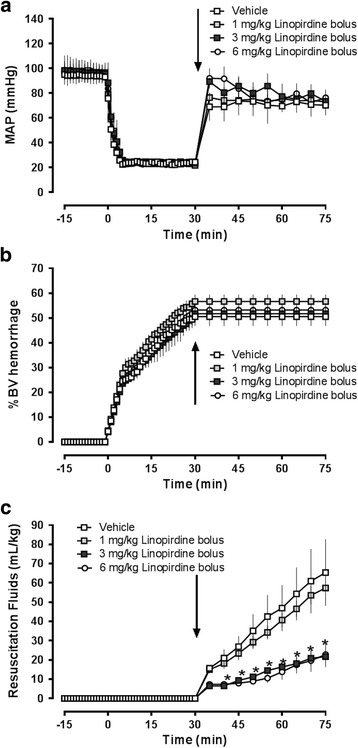
Linopirdine reduces resuscitation fluid requirements after hemorrhagic shock. Animals were hemorrhaged to a mean arterial blood pressure (MAP) of 25 mmHg for 30 min, followed by fluid resuscitation to a MAP of 70 mmHg. At the end of the shock period (t = 30 min), vehicle (*n* = 4, *open squares*), 1 mg/kg (*n* = 5, *light grey squares*), 3 mg/kg (*n* = 3, *black squares*) or 6 mg/kg (*n* = 3, *open circles*) linopirdine in 0.5 mL normal saline were injected intravenously. *Arrows* indicate the time point of drug injection. *: *p* < 0.05 vs. vehicle. **a**. Mean arterial blood pressure (MAP, mmHg). **b**. Hemorrhage volume in % of total blood volume (%BV hemorrhage). **c**. Resuscitation fluid requirements (mL/kg) to maintain MAP at 70 mmHg

**Fig. 3:**
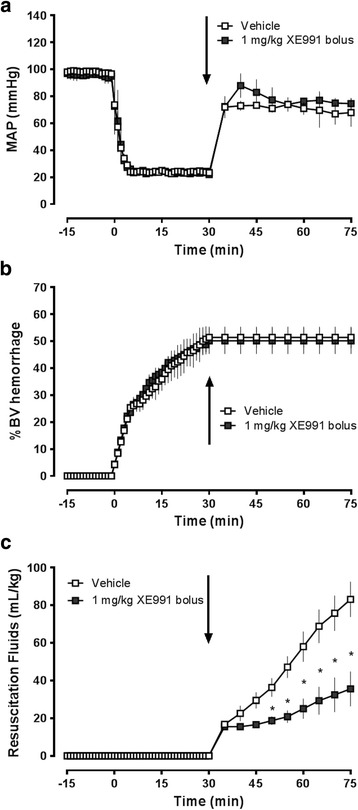
XE-991 reduces resuscitation fluid requirements after hemorrhagic shock. Animals were hemorrhaged to a mean arterial blood pressure (MAP) of 25 mmHg for 30 min, followed by fluid resuscitation to a MAP of 70 mmHg. At the end of the shock period (t = 30 min), vehicle (*n* = 3, *open squares*) or 1 mg/kg XE-991 (*n* = 3, *black squares*) in 0.5 mL normal saline were injected intravenously. *Arrows* indicate the time point of drug injection. *: *p* < 0.05 vs. vehicle. **a**. Mean arterial blood pressure (MAP, mmHg). **b**. Hemorrhage volume in % of total blood volume (%BV hemorrhage). **c**. Resuscitation fluid requirements (mL/kg) to maintain MAP at 70 mmHg

**Fig. 4:**
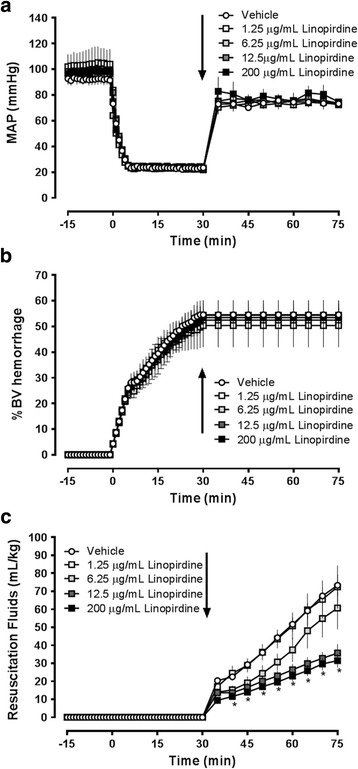
Supplementation of resuscitation fluids with linopirdine reduces resuscitation fluid requirements after hemorrhagic shock. Animals were hemorrhaged to a mean arterial blood pressure (MAP) of 25 mmHg for 30 min, followed by fluid resuscitation to a MAP of 70 mmHg. At the end of the shock period (t = 30 min), animals were resuscitated with normal saline (NS, vehicle, *n* = 4, *open circles*) or NS supplemented with 1.25 (*n* ﻿= 4﻿,open squares), 6.25 (*n* = 3, light grey squares), 12.5 (*n* = 3, dark grey squares) or 200 μg/mL (*n* = 6, black squares) linopirdine. *Arrows* indicate the time point of drug injection. *: *p* < 0.05 vs. vehicle. **a**. Mean arterial blood pressure (MAP, mmHg). **b**. Hemorrhage volume in % of total blood volume (%BV hemorrhage). **c**. Resuscitation fluid requirements (mL/kg) to maintain MAP at 70 mmHg

- Series 1: Administration of Kv7 channel modulators in normal animals.
- The effects of the Kv7 channel inhibitor linopirdine on arterial blood pressures in normal animals are shown in Fig. 1a. At baseline, MAP was 92 ± 2.5 mmHg. Within 5 min after intravenous linopirdine injection, MAP peaked at 92 ± 2 mmHg, 93 ± 2 mmHg, 95 ± 4 mmHg, 100 ± 2 mmHg and 105 ± 0.6 mmHg with linopirdine dosages of 0.1 mg/kg, 0.5 mg/kg, 1 mg/kg, 3 mg/kg and 6 mg/kg, respectively. Blood pressures returned to pre-injection values within ten to fifteen minutes. Figure 1b shows the blood pressure effects of the Kv7 channel activator retigabine. Intravenous retigabine injection dose-dependently reduced MAP from 91 ± 4 mmHg at baseline to 80 ± 8 mmHg with 0.5 mg/kg retigabine, and to 60 ± 5 mmHg, 42 ± 1 mmHg, 37 ± 1 mmHg and 34 ± 2 mmHg with 1 mg/kg, 3 mg/kg, 6mg/kg and 12 mg/kg retigabine, respectively. The duration of retigabine-induced hypotension increased with increasing doses. While MAP returned to pre-injection levels within 15 min when low doses of retigabine were injected (0.1 – 3 mg/kg), MAP did not recover to pre-injection values within 15 min at higher doses. Injection of 6 mg/kg linopirdine antagonized hypotension induced by 12 mg/kg retigabine.

- Series 2: Bolus administration of Kv7 channel inhibitors during resuscitation from hemorrhagic shock.
- We first tested the effects of single i.v. bolus injections of various doses of linopirdine after hemorrhagic shock. As shown in Fig. 2a/b, the blood volumes hemorrhaged to achieve a MAP target of 25 mmHg during the 30 min shock period was comparable in all animals. Linopirdine or vehicle were injected at the beginning of fluid resuscitation followed by crystalloid fluid resuscitation to maintain MAP at 70 mmHg. All animals could be resuscitated to the MAP resuscitation target. As compared with vehicle treated animals, linopirdine dose-dependently reduced fluid resuscitation requirements from 65 ± 34 mL/kg with vehicle to 57 ± 13 mL/kg, 22 ± 8 mL/kg (*p* < 0.05 vs. vehicle) and 22 ± 11 mL/kg (*p* < 0.05 vs. vehicle) with 1 mg/kg, 3 mg/kg and 6 mg/kg linopirdine, respectively (Fig. 2c). To test whether the resuscitation fluid-sparing effect of linopirdine can be generalized to other Kv7 channel inhibitors, we then compared the effects of XE-991 (1 mg/kg), a Kv7 channel inhibitor with higher potency than linopirdine, with vehicle treated animals under the same experimental conditions. The hemorrhage volumes were comparable between vehicle and XE-991 treated animals and all animals could be resuscitated to the MAP target (Fig. 3a/b). As observed for linopirdine, XE-991 significantly reduced cumulative resuscitation fluid requirements from 83 ± 16 mL/kg with vehicle treatment to 36 ± 16 mL/kg with XE-991 treatment (Fig. 3c).

- Series 3: Linopirdine-supplementation of resuscitation fluid.
- We then evaluated whether, instead of an i.v. bolus of linopirdine, supplementation of resuscitation fluids with various concentrations of linopirdine would also reduce cumulative resuscitation fluid requirements in the same model (Fig. 4). Similar to the previous experimental groups, hemorrhage volumes to achieve a MAP of 25 mmHg during the shock period were comparable between all groups and all animals could be resuscitated to a MAP of 70 mmHg. In this set of experiments, fluid resuscitation requirements were 73 ± 12 mL/kg with NS alone, 72 ± 24 mL/kg with NS supplemented with 1.25 μg/mL linopirdine and 61 ± 20 mL/kg when NS was supplemented with 6.25 μg/mL linopirdine (*p* > 0.05 vs. NS alone). When NS was supplemented with 12.5 μg/mL and 200 μg/mL linopirdine, however, resuscitation fluid requirements were reduced to 36 ± 9 mL/kg (*p* < 0.05 vs. NS alone) and 31 ± 9 mL/kg (*p* < 0.05 vs. NS alone), respectively.

We did not observe any macroscopic abnormalities, such as signs of ischemia, hemorrhage or infection, in any abdominal or thoracic organ during gross necropsies of the animals.

## Discussion

In the present study, we provide initial pre-clinical evidence suggesting that Kv7 channel inhibitors could be used to stabilize blood pressure and reduce fluid resuscitation requirements after hemorrhagic shock. Our findings on the blood pressure effects of the Kv7 channel inhibitor linopirdine and the Kv7 channel activator retigabine in normal Sprague-Dawley rats are in agreement with previous observations in the same strain of rats and confirm that both drugs dose-dependently modulate systemic blood pressure in vivo [18, 22]. In addition, our findings demonstrate that even a high intravenous bolus dose of linopirdine (6 mg/kg) and a total intravenous dose of 10.6 mg/kg of linopirdine administered within 1 h caused only minimal and short-lived increases in systemic blood pressures. In combination with the observation that intravenous injection of comparable doses of linopirdine to male Wistar rats did not affect systemic blood pressure [23], these data suggest that systemic vasopressor effects of linopirdine are consistently small. Despite only modest effects of acute linopirdine treatment on blood pressure in normotensive rats, we observed that retigabine-induced hypotension was instantaneously reverted with intravenous linopirdine. This finding is in agreement with urodynamic effects of retigabine and linopirdine [24] and with the effects of linopirdine on hypotension induced by kynurenine, a natural tryptophan metabolite that is thought to activate Kv7 channels and which has been implicated in the pathophysiology of sepsis [25]. As such, Kv7 channel modulators may provide an alternative pharmacological approach for the management of hypertensive emergencies, in which drugs that permit rapid, titratable and reversible reduction of blood pressure are highly desirable.

We detected that a single dose of linopirdine at the beginning of fluid resuscitation from hemorrhagic shock dose-dependently reduced fluid requirements to stabilize blood pressure. The observed effects of linopirdine were saturated at a dose of 3 mg/kg and resulted in 65% reduction of resuscitation fluid requirements. The observation that a dose of 1 mg/kg of the linopirdine analogue XE-991 was equally efficacious to reduce fluid resuscitation requirements as a dose of 3 mg/kg of linopirdine is consistent with the higher in vitro and in vivo potency of XE-991 [26]. These findings suggest that the fluid-sparing effects of linopirdine and XE-991 during resuscitation from hemorrhagic shock can be considered as a general pharmacological property of drugs that block Kv7 currents.

In an effort to optimize the dosing regimen, we then tested resuscitation fluids supplemented with various concentrations of linopirdine. As observed after single bolus injection, linopirdine also dose-dependently reduced fluid requirements when used as a resuscitation fluid supplement. The fluid-sparing effects of resuscitation fluid supplemented with 12.5 μg/mL linopirdine were comparable with the effects of a single 3 mg/kg bolus injection of linopirdine at the beginning of fluid resuscitation. Despite the similar efficacy of both dosing regimens to reduce resuscitation fluid requirements, supplementation of resuscitation fluid with linopirdine reduced the cumulative linopirdine dose that was administered during the experiment 7-*fold* to 0.45 mg/kg, a dose of linopirdine that has no noticeable effects on hemodynamics. Safety pharmacology testing of linopirdine has previously been performed in young and elderly volunteers, who received oral doses of up to 55 mg [27]. Furthermore, linopirdine has been orally administered in previous clinical trials in doses of 30 – 40 mg three times per day for up to six months [15, 28]. Although adverse effects on vital parameters were not noted in these studies, elevated alanine transferase levels have been described during oral administration of linopirdine [15]. Information on the effects of linopirdine after intravenous administration in humans, however, is currently not available. As Kv7 channels are abundantly expressed in numerous vascular beds and tissues [10, 16], further studies on possible side effects after intravenous linopirdine treatment are necessary. Nevertheless, we did not observe toxicity associated with linopirdine treatment in the present study, which is in agreement with previous observations after intravenous linopirdine administration in rodents [22, 24]. After oral administration in humans, the half-life of linopirdine is 0.4 – 3.2 h [27]. After intravenous injection of 2.5 mg/kg linopirdine, a half-life of 0.6 h has been determined in rats [29]. Thus, the short half-life would make linopirdine a drug that is easily controllable if adverse effects would occur.

As the present pilot study was designed to provide initial pre-clinical evidence for a possible new indication for the use of Kv7 channel blockers, we did not address the in vivo mechanisms leading to reduced resuscitation fluid requirements after hemorrhagic shock with Kv7 channel blockade. Nevertheless, one possible explanation is that Kv7 channel blockade could sensitize vascular smooth muscle function upon exposure to endogenous vasoconstrictors during the cardiovascular stress response after hemorrhagic shock. Alternatively, Kv7 channel blockade could reduce third-spacing of fluids during resuscitation from hemorrhagic shock. Mechanistic in vivo studies will be required to dissect the underlying mechanisms in the future.

## Conclusions

In conclusion, our findings point towards Kv7 channel inhibition as a new pharmacological approach to stabilize hemodynamics and reduce fluid resuscitation requirements after hemorrhagic shock. Although the short resuscitation period limits the scope of our pilot study, our observations provide proof of principle for a new indication for Kv7 channel blockers and initial information on the dose-effect relationship for linopirdine. The findings from the present study justify a more detailed pre-clinical evaluation of the therapeutic efficacy and possible side-effect profile of linopirdine after traumatic and hemorrhagic shock over longer time periods. Such studies on the re-purposing of linopirdine for the treatment of trauma patients could lead to a rapid transition into the clinical arena.

## Abbreviations

%BV: % of total blood volume
BP: Blood pressure
DBP: Diastolic arterial blood pressure
HR: Heart rate
MAP: Mean arterial blood pressure
NS: Normal saline
SBP: Systolic arterial blood pressure
SD: Standard deviation
